# Simultaneous Accumulation of Holocellulose, Callose and Lignin: Cell Wall Markers for Resistance in Wheat Infested with *Diuraphis noxia*

**DOI:** 10.3390/ijms26209874

**Published:** 2025-10-10

**Authors:** Siphephelo N. N. Zondo, Lintle Mohase, Vicki Tolmay, Mpho Mafa

**Affiliations:** 1 Carbohydrates and Enzymology Laboratory (CHEM-LAB), Department of Plant Sciences, University of the Free State, Bloemfontein 9300, South Africa; 2019416987@ufs4life.ac.za; 2 Department of Plant Science, Faculty of Natural and Agricultural Sciences, University of the Free State, Bloemfontein 9300, South Africa; mohasel@ufs.ac.za; 3 Agricultural Research Council-Small Grain Institute, Bethlehem 9700, South Africa; tolmayv@arc.agric.za

**Keywords:** Russian wheat aphid, cell wall reinforcement, holocellulose, callose, non-penetration papillae, lignification

## Abstract

Exposure of the plant cell wall to biotic and abiotic stresses results in structural and chemical changes. Russian wheat aphid (RWA) infestation severely damages wheat plants, releasing cell wall-degrading enzymes that compromise cell wall integrity. This study aims to elucidate the cell wall modifications in resistant wheat cultivars during RWA infestation. Three wheat cultivars with distinct resistance phenotypes to the RWA South African biotype 2 (RWASA2) were grown in the glasshouse. At the three-leaf stage, the seedlings were infested with RWASA 2 for 14 days. The leaf samples harvested at 2, 7, and 14 days post-infestation (dpi) were used to study cell wall modifications in the RWASA 2-infested cultivars, focusing on cellulose, hemicellulose, callose, and lignin contents. The results showed that post-RWASA2 infestation, the resistant Tugela-*Dn5* significantly increased the hemicellulose content by 2.8- and 1.3-folds at 2 and 14 dpi, respectively, while the Tugela and Tugela-*Dn1* significantly decreased the hemicellulose content at 2, 7, and 14 dpi. Tugela-*Dn5* also increased the cellulose content by 1.4-fold and 2.2-fold at 7 and 14 dpi, respectively. The acid-soluble lignin content significantly increased in the infested Tugela-*Dn5* compared to uninfested at 2 and 14 dpi, while it significantly decreased in Tugela and Tugela-*Dn1*. Callose levels also increased in all cultivars at 2 dpi, but only the infested Tugela-*Dn5* exhibited an increase in callose content compared to the uninfested at 14 dpi. The extracted contents of the increased cellulose, hemicellulose, and lignin in Tugela-*Dn5* were corroborated by FTIR analysis, which showed broad peaks at 3300 cm^−1^ representing the OH functional group and inter- and intra-hydrogen bonds within the increased cellulose in Tugela-*Dn5*. No significant reduction of lignin peaks at 1600 to 1578. 99 cm^−1^ assigned to the phenolic groups was observed in Tugela-*Dn5*. These findings place cell wall modifications at the centre of the wheat’s physiological resistance response to aphid infestation, particularly the reinforcement of the cell wall that persists for 14 dpi.

## 1. Introduction

The plant cell wall plays a vital role in plant growth, development, maintaining cell shape, and protecting the cell against various external stimuli [1,2,3]. The cell wall is damaged by exposure to biotic and abiotic stress, resulting in structural and chemical changes [2,3]. The major components of the cell wall include lignin, cellulose, and hemicellulose, with trace amounts of pectin, ash and protein [4]. In the case of wheat seedlings, Mafa et al. [5] showed that acid-soluble lignin constituted about 7%, while holocellulose content was about 60% of the cell wall components. The three major cell wall components, i.e., cellulose, hemicellulose and lignin, were used to demonstrate the impact of biotic and abiotic stresses on grain crops’ plant cell wall integrity [5,6].

The Russian wheat aphid (RWA: *Diuraphis noxia*) severely damages barley and wheat plants because it affects cell wall integrity during feeding [7,8]. Lapitan et al. [9] demonstrated that the protein extracts from the RWA indirectly compromised the structural integrity of the cell wall by inducing symptoms associated with susceptibility, including leaf rolling. During feeding, RWA secretes saliva containing cell wall-degrading enzymes (CWDE) that degrade the cell wall, which affects cell wall structural integrity by distorting the holocellulose and lignin contents in the susceptible cultivars [5,10]. The RWA saliva-associated CWDEs include enzymes such as pectinases, cellulases, hemicellulases, and β-1,3-glucanases, which respectively degrade pectin, cellulose, hemicellulose, and callose [5,11,12,13,14,15]. The breaching of the cell wall defence is associated with plant susceptibility and yield loss that could threaten food security because wheat is a stable food source for many countries globally.

The RWA evolved a complex cocktail of CWDEs due to cell wall polysaccharides with diverse chemical compositions, requiring specific enzymes for successful hydrolysis. For example, cellulose is composed of β-1,4-glucopyranoside units in a backbone chain, while monocots’ hemicellulose is a heterologous polysaccharide made of mixed-linked β-1,3-1,4-glucan, xylan (divided into three types: arabinoxylan, glucuronoxylan and xyloglucan) [14,15,16,17]. Lignin is resistant to glycoside hydrolases (GHs) enzymatic degradation [4], thus enhancing cell wall integrity and strength as part of an adequate protection strategy against pest/pathogen invasion [18]. It was proposed that reduced holocellulose and lignin contents in RWA-infested wheat plants indicated cell wall degradation in the susceptible cultivar. In contrast, increased components in the resistant cultivar formed part of the defence responses linked to resistance, i.e., cell wall reinforcement [5,18].

Previous studies identified various defence responses associated with cell wall modification. For example, callose deposition, which is associated with resistance during plant-pathogen interactions [19], was linked to susceptibility during wheat/barley-RWA interaction [7,8,12]. However, only a few studies have investigated the involvement of cell wall modifications (loosening or reinforcements) during the induction of host defence to RWA feeding in monocots such as wheat. Mafa et al. [5] demonstrated that increased holocellulose and lignin composition in resistant wheat cultivars was associated with reinforced cell walls, thus deterring RWA feeding. However, Vaahtera et al. [1] proposed a focus on understanding the mode of action of the plant cell wall structural integrity maintenance mechanism. Paying attention to various cell metabolic processes relevant to cell wall formation and modification, including complex cell wall features like cellulose, hemicellulose and lignin production during adaptive responses to RWA feeding, could be a crucial approach to understanding resistance response. Thus, the current study aimed to investigate how three wheat cultivars, i.e., Tugela (susceptible), Tugela-*Dn1* (moderately resistant) and Tugela-*Dn5* (resistant), modify their cell walls to reduce the effect of RWA probing and survive under infestation for a 14-day period. To achieve the aim, the experiments were performed to quantify the cell wall carbohydrates and acid-soluble lignin contents, followed by the FTIR analysis to quantify cell wall chemical changes, and callose deposition quantification after RWASA2 feeding.

## 2. Results

### 2.1. Hemicellulose and Cellulose Determination

**Figure 1:**
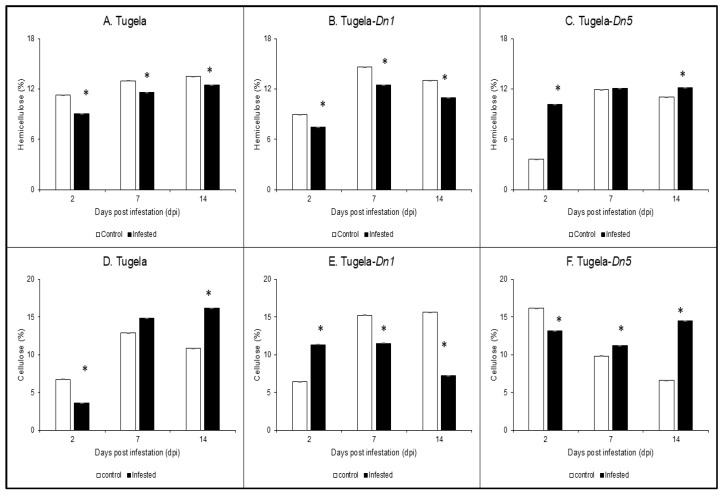
Analysis of hemicellulose (**A**–**C**) and cellulose (**D**–**F**) content in cell walls of Tugela, Tugela-*Dn1*, and Tugela-*Dn5* after infestation by RWASA2 for 2, 7, and 14 days. Control represents uninfested samples, and experiments were conducted in biological triplicate. The mean ± SD represents the values and error bars. ANOVA was used to calculate the significant differences between the treatments, and the *p*-value was set at *p* ≤ 0.05. The Tukey HSD post-hoc test (*p* ≤ 0.05) was performed to determine the significant differences represented by the asterisk (*) within the infested and uninfested.

The hemicellulose content of Tugela, Tugela-*Dn1*, and Tugela-*Dn5* was extracted with an alkaline solution, followed by the determination of hemicellulose using the phenol and sulfuric method. The results showed that RWASA2 infestation significantly (*p* ≤ 0.05) reduced the hemicellulose content in susceptible Tugela and moderately resistant Tugela-*Dn1* compared to uninfested samples from 2 to 14 dpi (Figure 1A,B). In contrast, resistant Tugela-*Dn5* displayed significantly (*p* ≤ 0.05) increased hemicellulose content upon RWASA2 infestation relative to uninfested at 2 and 14 dpi (Figure 1C). At 2 dpi, the RWASA2-infested Tugela-*Dn5* showed a more than 2.7-fold increase in the hemicellulose content (10.13%) compared to uninfested samples (3.6%), suggesting that it could be involved in the early defence responses. These findings showed a significant (*p* ≤ 0.05) reduction in the hemicellulose content of Tugela (9%, 11.58%, and 12.45%) compared to the uninfested (11.25%, 13.0%, and 13.51%) at 2, 7, and 14 dpi, respectively. Tugela *Dn1*, a moderately resistant cultivar, showed a similar response by significantly reducing the hemicellulose content from 2 to 14 dpi (Figure 1B).

Cellulose content was extracted using the sulphuric acid extraction procedure described by NREL [20], followed by detection using the phenol-sulfuric acid method. The results revealed that RWASA2-infested susceptible Tugela and resistant Tugela-*Dn5* cellulose content was significantly (*p* ≤ 0.05) reduced at 2 dpi, followed by an increased cellulose content at 7 and 14 dpi compared to uninfested (Figure 1D,F). In addition, cellulose content in RWASA2-infested Tugela gradually increased over the infestation time, while in Tugela-*Dn5* it decreased by 1.2-fold at 7 dpi, followed by a 2.19-fold increase at 14 dpi. Tugela-*Dn1* responded to RWASA2 infestation by significantly (*p* ≤ 0.05) increasing the cellulose content compared to uninfested at 2 dpi. However, at 7 and 14 dpi, this cultivar significantly (*p* ≤ 0.05) reduced the cellulose content in infested cultivars compared to uninfested (Figure 1E). These results revealed that the cell walls of the three wheat cultivars responded differently towards RWASA2 infestation. For instance, Tugela-*Dn5* and Tugela cellulose content showed a similar increasing pattern during the infestation period (Figure 1D,F), while Tugela-*Dn1* had a decreasing cellulose content over this period. The cellulose and hemicellulose results further confirmed that the Tugela and Tugela-*Dn1* cell walls responded differently from Tugela-*Dn5* at the hemicellulose level.

### 2.2. Total Holocellulose Determination

**Figure 2:**
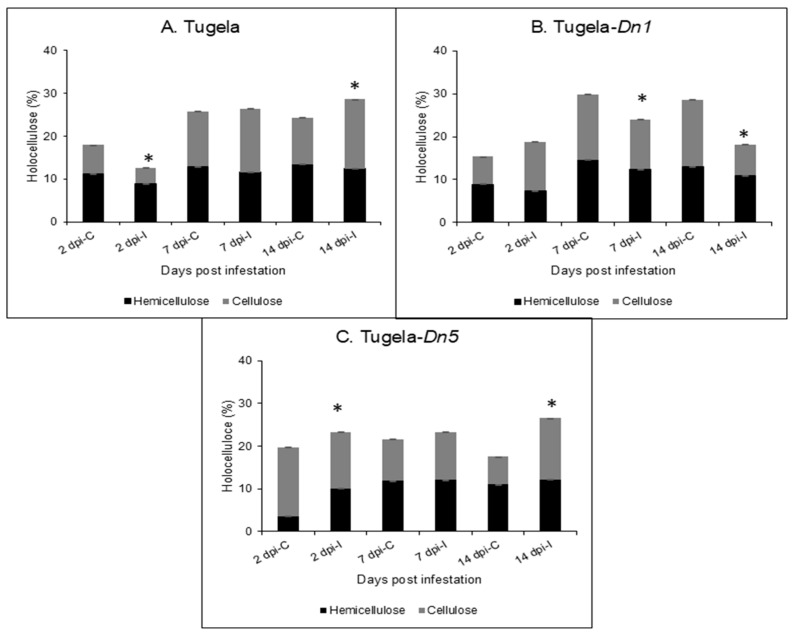
Analysis of the holocellulose content in the cell wall of Tugela (**A**), Tugela-*Dn1* (**B**), and Tugela-*Dn5* (**C**) after 2, 7, and 14 days post-RWASA2 infestation. Control represents the uninfested samples, and experiments were conducted in biological triplicate. The mean ± SD represents the values and error bars. ANOVA was used to calculate the significant differences between the treatments, and the *p*-value was set at *p* ≤ 0.05. The Tukey HSD post-hoc test (*p* ≤ 0.05) was performed to determine the significant differences represented by the asterisk (*) within the infested and uninfested treatments.

The holocellulose content of the cell wall is composed of cellulose and hemicellulose. It is generally used to understand how the major polysaccharide content of the plant cell wall responds to stress, and here, the holocellulose was used to understand how the cell wall responded to RWASA2 infestation. The Tugela cultivar had reduced holocellulose content at 2 dpi compared to the uninfested, followed by equal amounts at 7 dpi and an increase at 14 dpi (Figure 2A). In contrast, Tugela-*Dn1* showed an increased holocellulose at 2 dpi, which was followed by lower content in the infested leaf tissue at 7 and 14 dpi (Figure 2B). Interestingly, the holocellulose content in the resistant Tugela-*Dn5* increased over all the test time points after RWASA2 infestation compared to uninfested (Figure 2C). The results suggest that the decreased holocellulose content of the Tugela (2 dpi) and Tugela-*Dn1* (7 and 14 dpi) shows that the cell wall was potentially weakened during RWASA2 infestation. At the same time, Tugela-*Dn5* increased holocellulose content, suggesting the cell wall was fortified to resist further RWASA2 feeding.

### 2.3. Acid-Soluble Lignin Content

**Figure 3:**
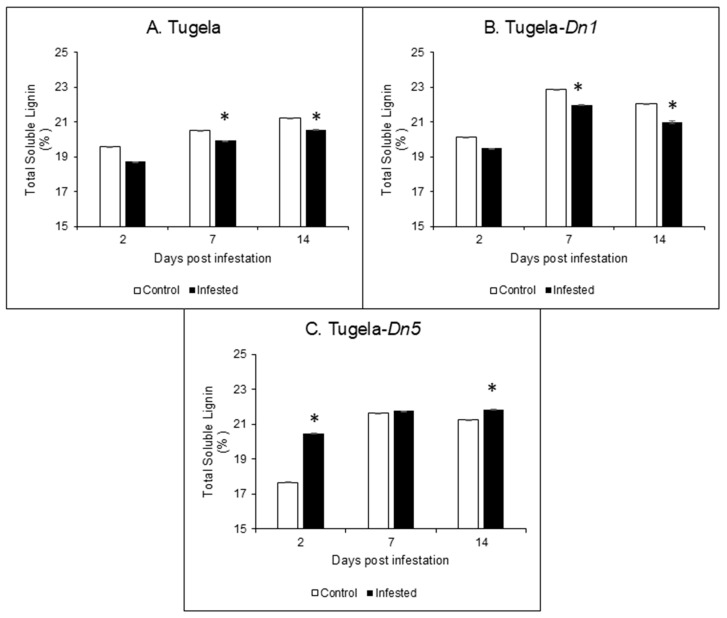
Analysis of the soluble lignin content in the cell walls of Tugela (**A**), Tugela-*Dn1* (**B**), and Tugela-*Dn5* (**C**) after 14 days of RWASA2 infestation. Control represents the uninfested samples, and experiments were conducted in biological triplicate. The values and error bars represent the mean ±SD. ANOVA was used to calculate the significant differences between the treatments, and the *p*-value was set at *p* ≤ 0.05. The Tukey HSD post-hoc test (*p* ≤ 0.05) was performed to determine the significant differences represented by the asterisk within the infested and uninfested treatments.

Soluble lignin was significantly (*p* ≤ 0.05) reduced in the susceptible Tugela and moderately resistant Tugela-*Dn1* cultivars post-RWASA2 infestation (Figure 3A,B), while infestation in the resistant Tugela-*Dn5* induced an increase in soluble lignin over 14 days compared to uninfested samples (Figure 3C). The soluble lignin in Tugela increased from 2 to 14 dpi, while in Tugela-*Dn1* it increased from 2 to 7 dpi, followed by a decrease at 14 dpi. However, the lignin in Tugela-*Dn5* increased over time, but there was no significant (*p* ≤ 0.05) difference in lignin content of infested samples at 7 and 14 dpi. These results demonstrated that the cell wall’s lignin content was reduced in the susceptible Tugela and moderately resistant Tugela-*Dn1* at 2, 7, and 14 dpi. In contrast, RWASA2 infestation increased the lignin content in the resistant Tugela-*Dn5* cultivar.

### 2.4. Fourier-Transform Infrared (FTIR) Spectroscopy Analysis

**Figure 4:**
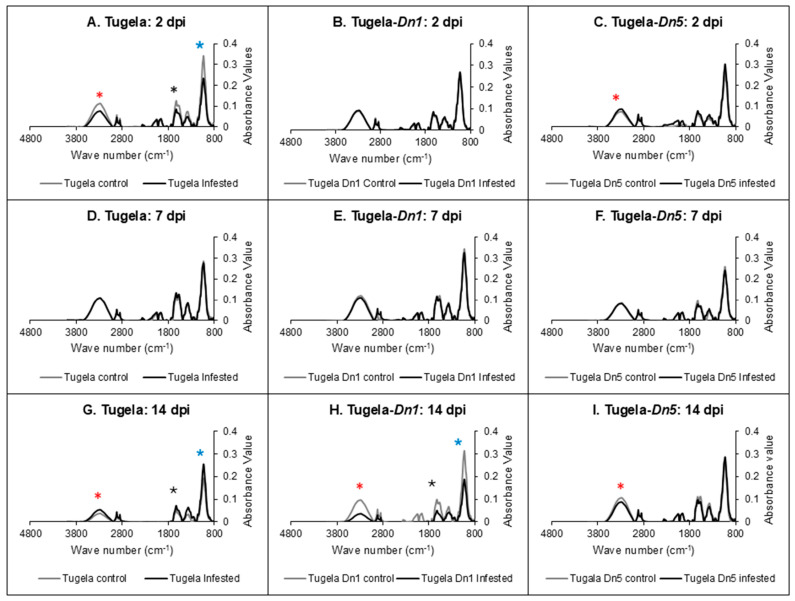
FTIR spectroscopy profiles of susceptible Tugela, moderate resistant Tugela-Dn1 and resistant Tugela-Dn5 cell walls (**A**–**I**). The spectra demonstrate the changes in the absorbance of functional groups in the cell walls of the RWASA2-infested and uninfested wheat cultivars at 2, 7, and 14 days post-infestation. Keys: * = cellulose region (3400-3000 cm^−1^); * = holocellulose region (1019 cm^−1^); * = lignin region (1600-1500 cm^−1^).

The FTIR was used to validate the changes observed in cell wall modification because of the holocellulose and lignin content changes in three wheat cultivars after RWASA2 infestation. FTIR also provided valuable insights into the chemical and structural changes in RWASA2-infested wheat cultivars’ cell walls compared to the uninfested. Figure 4 shows that RWASA2 infestation affected the holocellulose and lignin contents in the cell wall of Tugela at 2 dpi; Tugela-*Dn5* responded to RWASA2 infestation by increasing the microcrystalline cellulose and holocellulose content, while Tugela-*Dn1* showed no difference between uninfested and infested samples at these regions. The cell wall modifications in Tugela at 2 dpi were confirmed by a broad peak between 3400 and 3000 cm^−1^ with the highest point at 3300 cm^−1^ representing the OH functional group, associated with inter- and intra-hydrogen bonds within the microcrystalline cellulose region of the cell wall. Also, the peaks at 2915.59 and 2911.62 cm^−1^ were attributed to cellulose’s C–H bonds (stretching), were slightly reduced. In addition, the reduced lignin content in infested Tugela samples compared to uninfested samples was confirmed by the reduced peak at 1578.99 cm^−1^, assigned to the C=O carbonyl functional group of the aromatic rings of lignin. The 1600 cm^−1^ peak assigned to the phenolic or aromatic compounds was also reduced in the infested Tugela (Figure 4A). The highest peak at 1019.24 cm^−1^ was assigned to the glycosidic linkages (ether bond) found in the holocellulose region of the cell wall. The glycosidic linkages peak (1019.24 cm^−1^) was reduced by RWASA2 infestation in the susceptible Tugela compared to the uninfested. It is interesting to note that the crystalline cellulose peak (3300 cm^−1^) and the holocellulose peak (1019.24 cm^−1^) were slightly increased in the RWASA2-infested resistant Tugela-*Dn5* cultivar at 2dpi (Figure 4C).

The three Tugela cultivars infested with RWASA2 did not show increased cell wall modification at 7 dpi (see details in Figure 4D–F). However, at 14 dpi, the moderately resistant Tugela-*Dn1* showed reduced peaks for crystalline cellulose (3300 cm^−1^), phenolics (1600 cm^−1^), carbonyl functional group of the aromatic rings (1578 cm^−1^), and holocellulose (1019 cm^−1^) compared to uninfested samples (Figure 4H). The infested Tugela-*Dn5* only showed a slight reduction in the crystalline cellulose region (3300 cm^−1^) (Figure 4I). In contrast, RWASA2-infested Tugela had increased crystalline cellulose and holocellulose contents relative to the uninfested at 14 dpi (Figure 4G). These findings confirm that RWASA2 infestation triggered cell wall reinforcement during early feeding in the moderately resistant and resistant Tugela cultivars. The FTIR results also supported the holocellulose and lignin content results observed during the interaction of RWASA2 and Tugela or Tugela-*Dn5* in Figure 2 and Figure 3.

### 2.5. Callose Quantification in the RWASA2-Infested Wheat Cultivars

**Figure 5:**
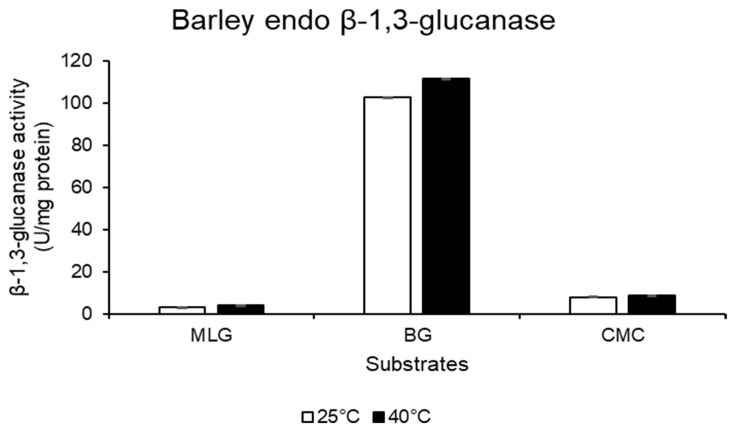
Barley β-1,3-glucanase substrate specificity analysed on mixed-linked β-1,3-1,4-glucan (MLG), β-1,3-glucan (curdlan: BG), and β-1,4-glucan (AZO-carboxymethyl cellulose: CMC) substrates. Reactions were conducted at 25 and 40 °C. The experiments were conducted in technical triplicate. The mean ± SD represents the values and error bars.

**Figure 6:**
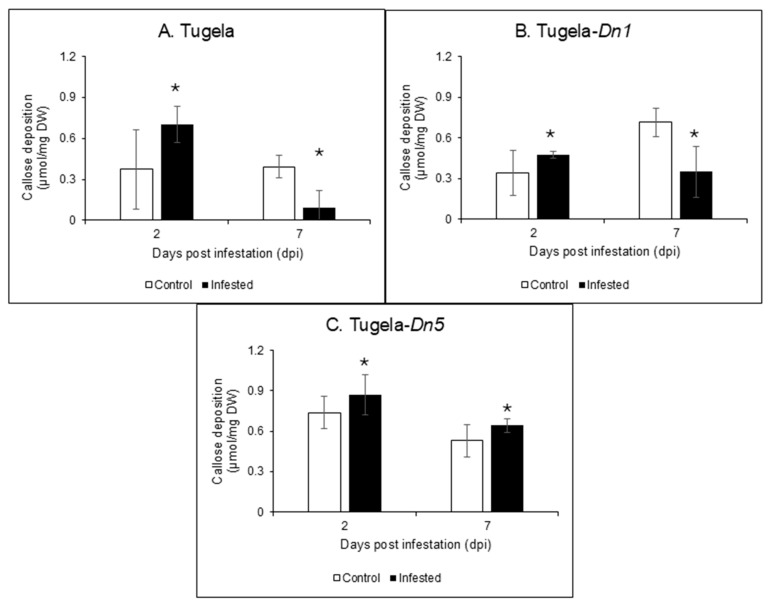
Analysis of callose content in the cell wall biomass of Tugela (**A**), Tugela-*Dn1* (**B**), and Tugela-*Dn5* (**C**) after 2 and 7 days of RWASA2 infestation. Control represents the uninfested treatment, and experiments were performed in biological triplicate. The values and error bars represent means ± SD, respectively. ANOVA was used to calculate the significant differences between the treatments, and the *p*-value was set at *p* ≤ 0.05. The Tukey HSD post-hoc test (*p* ≤ 0.05) was performed to determine the significant differences represented by the asterisk within the infested and uninfested treatments.

Callose (β-1,3-glucan) deposition in the cell wall of RWASA2-infested Tugela, Tugela-*Dn1*, and Tugela-*Dn5* was hydrolysed using commercial barley *endo*-β-1,3-glucanase (highly specific for β-1,3-glucan/callose; Figure 5). The enzyme hydrolysis products were measured by the DNS method to quantify the total reducing sugars, expressed as µmol/minute/mg protein, which represented the concentration of callose in the three wheat cultivars. Saheed et al. [8] influenced our sample selection for callose measurements (only 2 and 7 dpi samples), as they indicated that callose levels increased between 2 and 3 days after RWA infestation; while between 3 and 7 dpi, there was no increase in the callose level of the susceptible cultivar. The results indicated that RWASA2 infestation increased callose content in the cell walls of all three cultivars at 2 dpi (Figure 6). At 7 dpi, the callose content in the RWASA2-infested Tugela samples was significantly (*p* ≤ 0.05) reduced compared to the uninfested (Figure 6A). Similarly, the infested Tugela-*Dn1* samples increased the callose content at 2 dpi, followed by a significant reduction in callose content compared to the uninfested at 7 dpi (Figure 6B). Interestingly, the resistant Tugela-*Dn5* demonstrated increased callose levels at 7 dpi (0.64 µmol/mg dry weight) compared to the uninfested (0.53 µmol/mg dry weight). These findings indicate that all cultivars significantly induced the callose levels at 2 dpi, but the susceptible Tugela and moderately resistant Tugela-*Dn1* significantly reduced the callose levels at 7 dpi. In contrast, the resistant Tugela-*Dn5* cultivar increased the callose levels at 7dpi compared to the uninfested samples.

## 3. Discussion

The major cell wall polymers analysed in this study during RWASA2-wheat interactions were cellulose, hemicellulose, and lignin. During plant biotic stress, such as RWA feeding, the cell wall modifications form part of defence responses that protect tissues and cells from damage. Cell wall modification in the resistant wheat cultivars increases holocellulose and lignin content, reinforcing the cell wall structural integrity to cope with biotic stress [5,6,21]. Cell wall reinforcement protects plants from aphids and other biotic stress agents by decreasing the effects of the CWDEs’ activity in the cell wall [22]. Previous studies demonstrated that callose accumulated in the susceptible wheat cultivar compared to the resistant one during RWA infestation [7,8,12,23]. Other studies argued that callose levels were lower in the resistant plants because of the formation of papillae composed of callose, holocellulose and lignin [5,24]. Also, the increased activity of β-1,3-glucanase is accompanied by callose degradation in resistant cultivars [25]. This study investigated how cell wall modification and callose levels in susceptible and resistant wheat cultivars infested with RWASA2 shed light on the role of cell wall reinforcement and papillae formation in defence responses.

Hemicellulose reinforces the cell wall by tethering cellulose microfibrils [22]. Hemicellulose and cellulose together form the holocellulose content. Several studies have suggested that the holocellulolytic content increases in resistant cultivars during pest and pathogen attacks [5,6,15,22], indicating that it is deposited in the cell wall region in response to biotic stress. The findings showed that RWA-wheat interaction significantly (*p* ≤ 0.05) enhanced the holocellulose content of the resistant Tugela-*Dn5*, whereas the susceptible Tugela delayed the increase of the holocellulolytic content up to 7 days during the initial infestation period. It is worth noting that moderate Tugela-*Dn1* increased the holocellulolytic content by 2 dpi; however, this content was reduced significantly (*p* ≤ 0.05) compared to uninfested samples at 7 and 14 dpi. These results indicated that cell wall reinforcement occurred throughout the infestation period in resistant Tugela-*Dn5*. For example, the moderately resistant cultivar responded to infestation by increasing the holocellulose content during early infestation periods (2 dpi), followed by a significant (*p* ≤ 0.05) reduction of holocellulolytic content compared to uninfested at the late infestation periods (7 and 14 dpi). In contrast, the holocellulose level significantly (*p* ≤ 0.05) decreased during early feeding (2 dpi) but increased at 14 dpi in susceptible cultivars. Therefore, delayed defence responses influence RWASA2 feeding at an early infestation period. Previous studies have demonstrated that holocellulose content increased in resistant wheat during the pest (RWA) or pathogen (leaf rust fungus) attack, while it is significantly (*p* ≤ 0.05) reduced in susceptible cultivars due to degradation by CWDE [5,6].

The presence of the lignin barrier protects the holocellulolytic content of the cell wall from cellulases and hemicellulases [26]. Furthermore, Mnich et al. [18] argued that lignin plays a crucial role in strengthening and reinforcing cell wall structure, particularly during biotic stress. The results indicated that the RWASA2-infested Tugela and Tugela-*Dn1* cultivars exhibited a reduction in lignin content compared to the uninfested samples during both early (2 dpi) and late (7-14 dpi) feeding periods. In contrast, the infested resistant Tugela-*Dn5* cultivar showed an increase in lignin content from 2 to 14 dpi. These findings corroborate previous research by Mafa et al. [5], demonstrating that increased lignin content is a strong marker for resistance against RWA infestation. Additionally, resistant wheat cultivars displayed significantly (*p* ≤ 0.05) higher POD activity during RWA infestation [25]. The increased POD activity and lignin content imply that lignification processes, which led to lignin crosslinking or lignin-hemicellulose cross-linking, occur in the resistant cultivar following RWASA2 infestation. Other studies also speculated about the marked increase in cell wall lignification after infestation [12,27]. The findings of the current study confirmed that lignification in resistant cultivars deterred RWASA2 feeding, while insufficient or suppressed lignification resulted in higher feeding rates in the moderately resistant and susceptible cultivars.

The findings of the extracted cell wall components post-RWASA2 infestation were validated with FTIR results. These results showed increased crystalline cellulose (3300 cm^−1^ peak), holocellulose (1018.76 cm^−1^) and lignin (1500–1730 cm^−1^) contents at 2 dpi in the resistant but slightly suppressed levels in the susceptible cultivars. Additionally, the FTIR profiles of Tugela, Tugela-*Dn1* and Tugela-*Dn5* at 7 dpi showed that the cell wall did not change relative to uninfested samples. At 14 dpi, it was interesting to observe that the Tugela crystalline cellulose, holocellulose and lignin absorbance values were increased significantly (*p* ≤ 0.05) relative to uninfested but were significantly (*p* ≤ 0.05) suppressed in Tugela-*Dn1*. These observations also confirmed that a delayed cell wall strengthening, or reinforcement response, was associated with the susceptible cultivar, while the early reinforcement and cell wall weakening responses at 14 dpi were observed in the moderately resistant cultivar. It is speculated that resistant cultivars had maintained cell wall strengthening and reinforcement during RWASA2-infestation. The information also indirectly demonstrated that the CWDEs in the RWA saliva could not easily degrade the cell wall in the resistant cultivar because it was strengthened and reinforced, early (2 dpi) during RWASA2 infestation. Several studies confirmed that RWA saliva or extracts could induce leaf rolling in the susceptible cultivars [9,28] or identified cellulase and hemicellulase enzymes in the RWASA2 and RWASA5 [5,29]. The significance of the cell wall strengthening through lignin cross-linking and reinforcement by cellulose and hemicellulose deposition is to reduce the impact of the CWDEs secreted by the RWASA during infestation.

The holocellulose, lignin (phenolics), and callose comprise the tough non-penetration papillae (NPP), while significantly (*p* ≤ 0.05) higher callose accumulation can lead to formation of the soft penetration papillae (PP) [15,30,31]. The formation of papillae has not been studied in detail during wheat-aphid interactions. However, several studies have demonstrated significantly (*p* ≤ 0.05) higher callose accumulation at the site of attack by aphids in susceptible cultivars [8,12]. The current study observed high callose deposition in the RWASA2-infested Tugela, Tugela-*Dn1*, and Tugela-*Dn5* at 2 dpi. However, at 7 dpi, the callose levels in Tugela and Tugela-*Dn1* were significantly (*p* ≤ 0.05) reduced compared to uninfested samples, while the Tugela-*Dn5* callose levels remained significantly (*p* ≤ 0.05) higher than in the uninfested samples. Saheed et al. [8] demonstrated that the susceptible cultivar released higher callose levels, detectable within 24 h, reaching a peak at 72 h; after 7 dpi, callose levels did not change until 14 dpi. Villada et al. [31] reported callose deposition within 20 min post-infestation associated with defence against aphids in resistant muskmelon (*Cucumis melo*). Susceptible *Zea mays* cultivars also exhibited less callose deposition following *Rhopalosiphum maidis* infestation [19]. Most studies on aphids (including RWA) have treated callose deposition separately, and those that investigated cell wall modification, i.e., lignification, also focused on this plant defence response as distinct entities. To understand the defensive or protective roles of non-penetration papillae (NPP) relative to penetration papillae (PP), as well as their function during RWA infestation, the callose, holocellulose, and lignin contents of RWA-infested wheat cultivars should be quantified simultaneously from the same samples, as suggested by Chowdhury et al. [15], who studied NPP and PP formation in barley infected with *Blumeria graminis f. sp. hordei*. The findings of the current study suggested that RWASA2-infested resistant cultivars induced the formation of NPP because throughout the infestation period, callose, holocellulose, and lignin contents were significantly (*p* ≤ 0.05) increased in the resistant cultivars. However, the reduced lignin and holocellulose contents in the infested Tugela compared to the uninfested samples at 2 dpi indicate possible PP formation. Even though callose was deposited promptly in this cultivar, the lack of lignin and holocellulolytic contents suggests that penetration papillae formed, based on Chowdhury’s theory [15].

## 4. Materials and Methods

### 4.1. Materials

Tugela, Tugela-*Dn1* and Tugela-*Dn5* seeds were obtained from the Agricultural Research Council–Small Grain (ARC-SG) Institute, Bethlehem, South Africa. The Tugela, Tugela-Dn1 and Tugela-Dn5 were selected for this study because they are susceptible, moderately resistant and resistant to the RWASA2 infestation as described by Jankielsohn [32]. A population of RWASA2 was allowed to multiply from apterous parthenogenetic female aphids on the susceptible PAN 3118 wheat cultivar in the glasshouse at the University of the Free State, Bloemfontein Campus (29°6′31.94″ S; 26°11′18.95″ E). The commercial barley *endo*-β-1,3-glucanase enzyme was purchased from Megazyme (Wicklow, Ireland). Unless stated otherwise, all the chemicals for buffer preparations and all analytical chemicals used in the study were purchased from Sigma (Johannesburg, South Africa).

### 4.2. Plant Growth Conditions

A split-plot design with four replicates was used for the trial, with infestation as the main plot and cultivars as sub-plots. Plants were cultivated according to Mafa et al. [5]. Briefly, 250 seeds of susceptible Tugela, moderately resistant Tugela-*Dn1* and resistant Tugela-*Dn5* cultivars were germinated in 15 separate Petri dishes. Each Petri dish contained 50 seeds. Germination conditions were as follows: 5 mL of distilled water was added to each Petri dish containing seeds. Petri dishes were sealed with parafilm and incubated in a dark germination chamber at 25 °C for 2 days. Tugela seeds differed from other seeds and took 7 days to germinate. The germinated seeds of each cultivar were transplanted into 15 cm pots containing 1:1 soil and peat moss (Mikskaar Professional Substrate) under controlled glasshouse conditions at the UFS, Bloemfontein Campus, South Africa. The plants were grown under a natural photoperiod and temperature regime (18 ± 2 °C at night and 24 ± 2 °C during the day). The plants were irrigated every three days using filtered tap water and fertilised with 2 g/L Multifeed classic (NULANDIS^®^; Edenvale, South Africa) [5.2.4 (43)] 14 days after the germinated seeds were transplanted into the pots. The second fertiliser application was performed 14 days after the first fertilisation. The plants were enclosed in cages covered with nets and allowed to grow to the second leaf-seedling stage.

### 4.3. Russian Wheat Aphid (RWASA2) Infestation

Tugela, Tugela-*Dn1*, and Tugela-*Dn5* wheat cultivars were infested with RWASA2 according to Mohase and Taiwe [28]. Twenty apterous parthenogenetic female aphids were used to infest each plant at the three-leaf growth stage. The uninfested and infested plants were kept in separate rooms. All plants were enclosed in cages covered with nylon nets (315 µm). There were two harvesting regimes: short-term feeding (2 days) and long-term feeding (7 and 14 days) post-infestation. At each harvesting time, ten randomly selected leaves (second and third) of each cultivar were cut at the base, wrapped in a moist paper towel, immediately placed on ice and transferred to the laboratory.

### 4.4. Total Carbohydrate Determination

Tugela, Tugela-*Dn1,* and Tugela-*Dn5* wheat cultivars were cultivated, infested with RWASA2 and harvested as described above. The leaf samples were ground into powder using liquid nitrogen before drying in the oven at 60 °C for 72 h. The dried samples were stored in an airtight container before use.

#### Hemicellulose, Cellulose and Lignin Content Quantification

A modified National Renewable Energy Laboratory (NREL) method [20] was employed to determine the cell wall composition of the RWASA2-infested and uninfested wheat cultivars. To avoid soluble sugars influencing the holocellulose content, they were removed using the method of Zhao et al. [33] before quantifying the cellulose, hemicellulose and lignin. Briefly, about 100 mg of the dried tissue samples were washed three times with 2 mL of 80% (*v*/*v*) ethanol and incubated at 80 °C for 15 min, followed by centrifugation at 3000× *g* for 10 min. The supernatant was discarded, and the leaf pellets were washed twice with 2 mL of distilled water and centrifuged at 10 000× *g* for 30 min. The pellets were used to quantify the cellulose, hemicellulose and lignin compositions in the wheat leaf.

The extraction of xylan (xylan represents the hemicellulose content in cereals) and soluble lignin from the leaf samples was performed using the modified alkaline extraction method by Gufe et al. [34]. Briefly, 1 mL of an alkaline sodium hydroxide solution [10% (*w/v*)] was added to washed leaf pellets, incubated at 60 °C for 3 h, with agitation every 30 min using a vortex. The mixture was centrifuged at 10,000× *g* for 30 min, and the supernatant containing hemicellulose and lignin mixture was collected and neutralised to pH 5 using 100% (*v/v*) glacial acetic acid. The remaining pellets were used to quantify the cellulose content in the leaf samples. The hemicellulose and soluble lignin were precipitated in ethanol by incubating overnight at 4 °C. The precipitate was collected by centrifugation at 13,000× *g* for 5 min.

The cellulose was completely hydrolysed with 72% (*w*/*w*) sulfuric acid by incubating at 30 °C for 1 h, followed by cooling on ice for 5 min [20]. After cooling, the mixture was diluted with 2× distilled water to 4% (*w*/*w*) sulfuric acid and autoclaved at 121 °C for 15 min. The hydrolysate was centrifuged at 13,000× *g* for 5 min and used to quantify the cellulose using a modified phenol-sulfuric acid protocol [35]. The hydrolysates were diluted twice (xylan and lignin) and five times (cellulose) before 1 mL aliquots of the diluted samples were transferred to clean 15 mL Falcon tubes, mixed with 1 mL of 5% (*w*/*v*) saturated phenol, followed by the addition of 2.5 mL concentrated sulfuric acid. The solutions were then incubated at 25 °C for 10 min before measuring the absorbance values at 490 nm for cellulose and hemicellulose, while soluble lignin was measured at 280 nm using a spectrophotometer (Genesys 180 UV-Visible Spectrophotometer, Thermo Fisher Scientific, Waltham, MA, USA). Glucose, xylose, and rutin were used as standards to calculate cellulose, hemicellulose, and soluble lignin compositions, expressed in percentages.

### 4.5. Fourier-Transform Infrared (FTIR) Spectroscopy Analysis of the Cell Wall Samples

RWASA2-infested and uninfested wheat cultivars were further analysed for cell wall physicochemical changes using FTIR, as described by [36]. Leaf samples were dried at 60 °C for 72 h, and about 1 mg of the dried sample was prepared to analyse the cell wall functional groups using an FTIR instrument (Thermo Scientific^TM^ Smart^TM^ iTX ATR, Thermo Fisher Scientific). It is important to note that the samples loaded on the FTIR sample holder were enough to cover the diamond crystal sample holder for better contact with a laser beam. The spectra were collected using the absorbance function (mode) at a resolution of 4.00 cm^−1^, with 32 scans per sample over the 4000 to 800 cm^−1^ spectrophotometer range. Spectra normalisation, manual baseline corrections, and peak integration were all performed using Omnic series software (v9.16). The FTIR micro-spectroscopic imaging system used was Nicolet iS20 FTIR Spectrometer (Thermo Fisher Scientific).

### 4.6. Callose Quantification in RWASA2-Infested Wheat Samples

The pure commercial barley *endo*-β-1,3-glucanase enzyme from Megazyme (Wicklow, Ireland) only has specific activity on β-1,3-glucan substrates (Figure 6). These substrates are similar to callose at chemical and structural levels because they comprise the glucose units’ backbone joined by β-1,3-glycosidic units. Based on its high specificity towards callose hydrolysis, barley *endo*-β-1,3-glucanase was used to hydrolyse and quantify the callose deposited in RWASA2-infested leaf cell wall biomass. The 2 and 7 dpi samples were used to quantify the callose because Saheed et al. [8] showed that callose was deposited incrementally from 1, 3 and 7 dpi, and no further increase was observed beyond 14 dpi. The reaction conditions to hydrolyse the callose from the leaf tissue of susceptible Tugela, moderately resistant Tugela-*Dn1*, and resistant Tugela-*Dn5* consisted of 1% (*w*/*v*) dried leaf tissue, which was hydrated with 50 mM sodium citrate buffer at pH 5. The *endo*-β-1,3-glucanase (purchased from Megazyme) dose was kept at 0.025 mg/mL, and the mixture was incubated at 40 °C for 24 h, followed by heating at 100 °C for 5 min to terminate the reaction. The reaction mixtures were centrifuged, followed by determining the total reducing sugars using the DNS reagent method by Miller [36]. Callose content was expressed in µmol/mg DW, where DW represents oven-dried weight.

### 4.7. Data Collection and Analysis

All experiments were randomised to avoid bias during infestation and sample collection, and all experimental analysis was conducted in triplicate. Microsoft Excel was used to analyse all the data and generate graphs (including standard curves). The values in the graphs generated from the collected experimental data represent the means ± standard error unless stated otherwise. Statistical analysis was performed with TIBCO Statistica (version 13.1), whereby multifactorial analysis of variance (ANOVA) was performed to test for significance between infested and uninfested treatments, and Tukey’s HSD was used to test for homogeneous groups at a *p*-value of 0.05.

## 5. Conclusions

The current study demonstrated that during wheat-RWASA2 interaction, resistant wheat cultivars reinforced the cell wall by increasing the holocellulose and lignin over infestation periods compared to the uninfested. The findings of the cell wall composition methods were validated with the FTIR analysis, confirming that the cell wall reinforcement was part of the defence responses against RWA infestation. In addition, callose levels were increased in Tugela-*Dn5* in response to RWASA2 infestation compared to uninfested. The simultaneous increase of callose, holocellulose, and lignin indicates that RWASA2-infested Tugela-*Dn5* could induce the formation of NPP. Unlike PP, which is mainly composed of callose, the NPP is composed of cellulose, hemicellulose, lignin and callose according to Chowdhury’s theory [15]. This study also argued that simultaneous cellulose, hemicellulose, lignin and callose accumulations could be used as a marker for cell wall strengthening in the resistant cultivar during RWA infestation. Tugela-*Dn5* significantly (*p* ≤ 0.05) increased lignin, hemicellulose, and callose content throughout the infestation periods, but all these components were significantly (*p* ≤ 0.05) decreased in the susceptible cultivar. In conclusion, the cell wall composition analysis of the RWASA2-infested wheat cultivars and the uninfested samples confirmed that the resistant cultivars responded by strengthening or reinforcing the cell wall. This study demonstrates the formation of the NPP in the wheat cultivars that are resistant to infestation, placing the cell wall modification at the centre of the resistant cultivar. Future work will focus on mechanisms that lead to the formation of the different NPP and PP in resistant and susceptible cultivars, respectively.

## Data Availability

The original contributions presented in this study are included in the article/supplementary material. Further inquiries can be directed to the corresponding author(s).

## Abbreviations

The following abbreviations are used in this manuscript:

ANOVA	Analysis of variance
ARC-SG	Agricultural Research Council–Small Grain Institute
cm^−1^	per centimetre
CWDE	Cell wall degrading enzymes
*Dn*	*Diuraphis noxia* resistance gene
DNS	3,5-Dinitrosalicylic acid reagent
dpi	Days post infestation
FTIR	Fourier Transform Infrared Spectroscopy
GH	Glycoside hydrolases
H	Hours
min	Minutes
NPP	Non-penetration papillae
NREL	National Renewable Energy Laboratory
OH	Hydroxylic group
PP	Penetration papillae
RWA	Russian wheat aphids
RWASA2	Russian wheat aphid South African biotype
